# Meconium Peritonitis, Intestinal Atresia Combined With Biliary Atresia: A Case Report

**DOI:** 10.3389/fped.2022.917116

**Published:** 2022-06-02

**Authors:** Yijiang Han, Shuqi Hu, Baohai Chen, Shoujiang Huang, Qi Qin, Jinfa Tou

**Affiliations:** ^1^Department of Neonatal Surgery, The Children's Hospital, Zhejiang University School of Medicine, National Clinical Research Center for Child Health, Hangzhou, China; ^2^Department of Information Center, The Children's Hospital, Zhejiang University School of Medicine, National Clinical Research Center for Child Health, Hangzhou, China

**Keywords:** meconium peritonitis, intestinal atresia, biliary atresia, short bowel, Kasai

## Abstract

Meconium peritonitis (MP) combined with intestinal atresia (IA) is a rare neonatal condition, and it is even rarer in combination with biliary atresia (BA). We describe a case of an infant who developed short bowel syndrome after partial intestinal resection due to MP and IA, along with a Santullienterostomy. During continuous enteral and parenteral nutrition, the stool color became paler. BA was identified by elevated direct bilirubin (DBIL), gamma-glutamyltransferase (GGT), serum matrix metalloproteinase-7 (MMP-7), and hepatobiliary ultrasound; then, Kasai portoenterostomy (KPE) was performed promptly. The Roux-en-Y limb was adjusted intraoperatively to preserve the maximum length of the small intestine while closing the enterostomy. After the operation, the infant gradually adapted to enteral nutrition, his bilirubin level returned to normal, and his weight gradually caught up to the normal range. Although rare, BA should be suspected when MP is combined with IA and when the stool becomes paler in color in the enterostomy state.

## Introduction

Meconium peritonitis (MP) is a rare neonatal condition in which small bowel atresia is one of the underlying causes and needs to be urgently identified for surgical intervention (1, 2). Combined with biliary atresia (BA), MP is even rarer, and early Kasai portoenterostomy (KPE) is essential for survival of the native liver and for the patient's survival (3). However, MP and IA sometimes require enterostomy. Intravenous nutrition can cause cholestasis, which presents difficulties in the differential diagnosis of BA. It is also not possible to apply the standard Roux-en-Y loop structure, which requires adjustment of the intestinal length to reconstruct the procedure. We report an infant with BA who developed short bowel syndrome after partial intestinal resection and Santullienterostomy for MP, associated with IA. A combination of methods was used to diagnose BA, and the length of the Roux-en-Y limb was adjusted during KPE. Postoperatively, the patient resumed total enteral nutrition and the cholestasis gradually subsided.

## Case Description

A male infant (G1P1, 39 + 3 weeks; birth weight, 3,430 g) was admitted to our neonatal unit immediately after birth with intestinal obstruction. Two months ago, an ultrasound examination revealed intestinal dilatation. Half a month ago, MRI suggested fetal meconium peritonitis combined with incomplete intestinal obstruction, and ascites was significantly less than before. Both parents were 25 years old, and neither reported a family history of gastrointestinal or liver disease. There was no maternal drug abuse during the entire pregnancy. She caught a cold in the 5th month of pregnancy and recovered quickly without medication.

**Figure 1:**
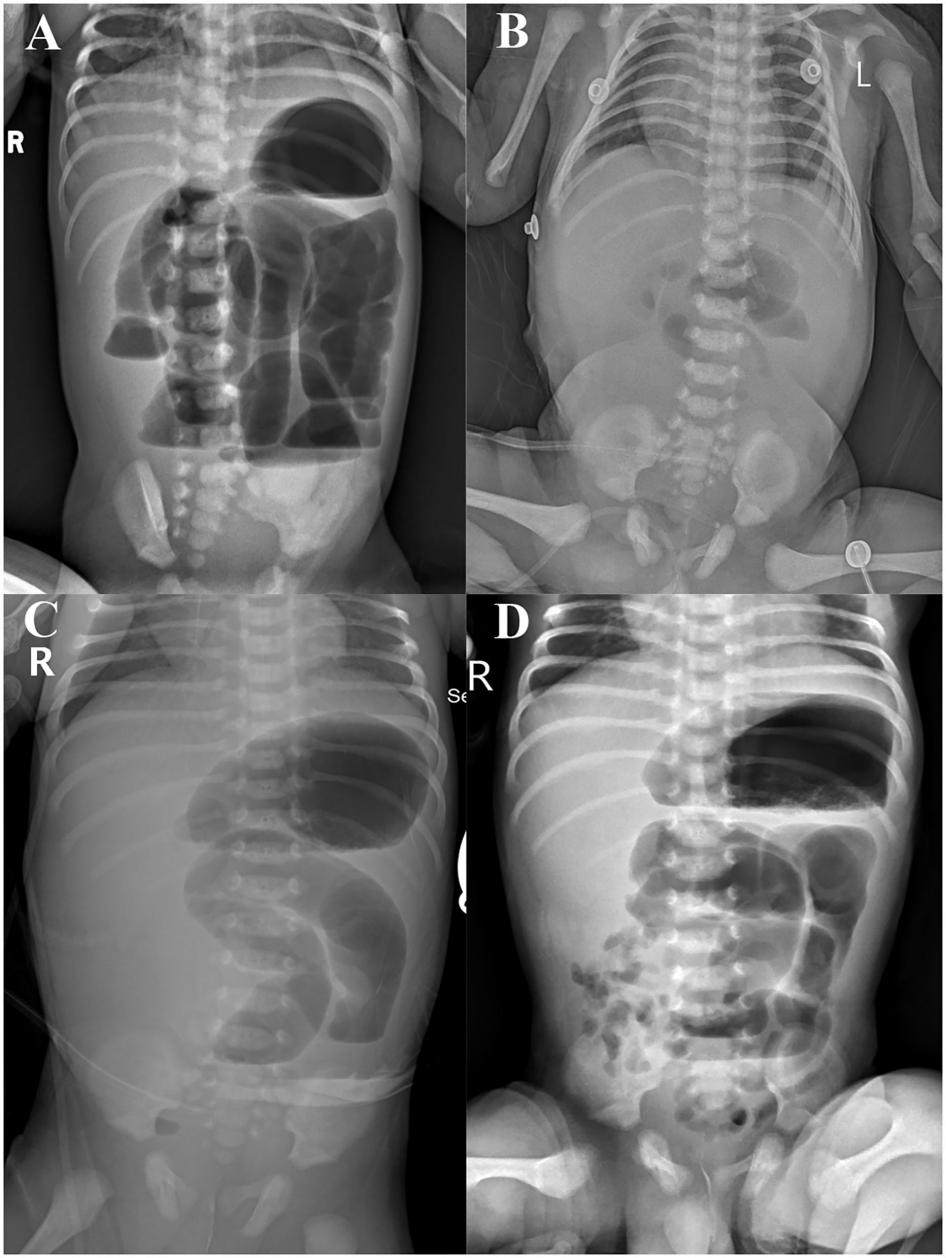
Abdominal x-rays before **(A)** and after **(B–D)** the first surgery.

**Figure 2:**
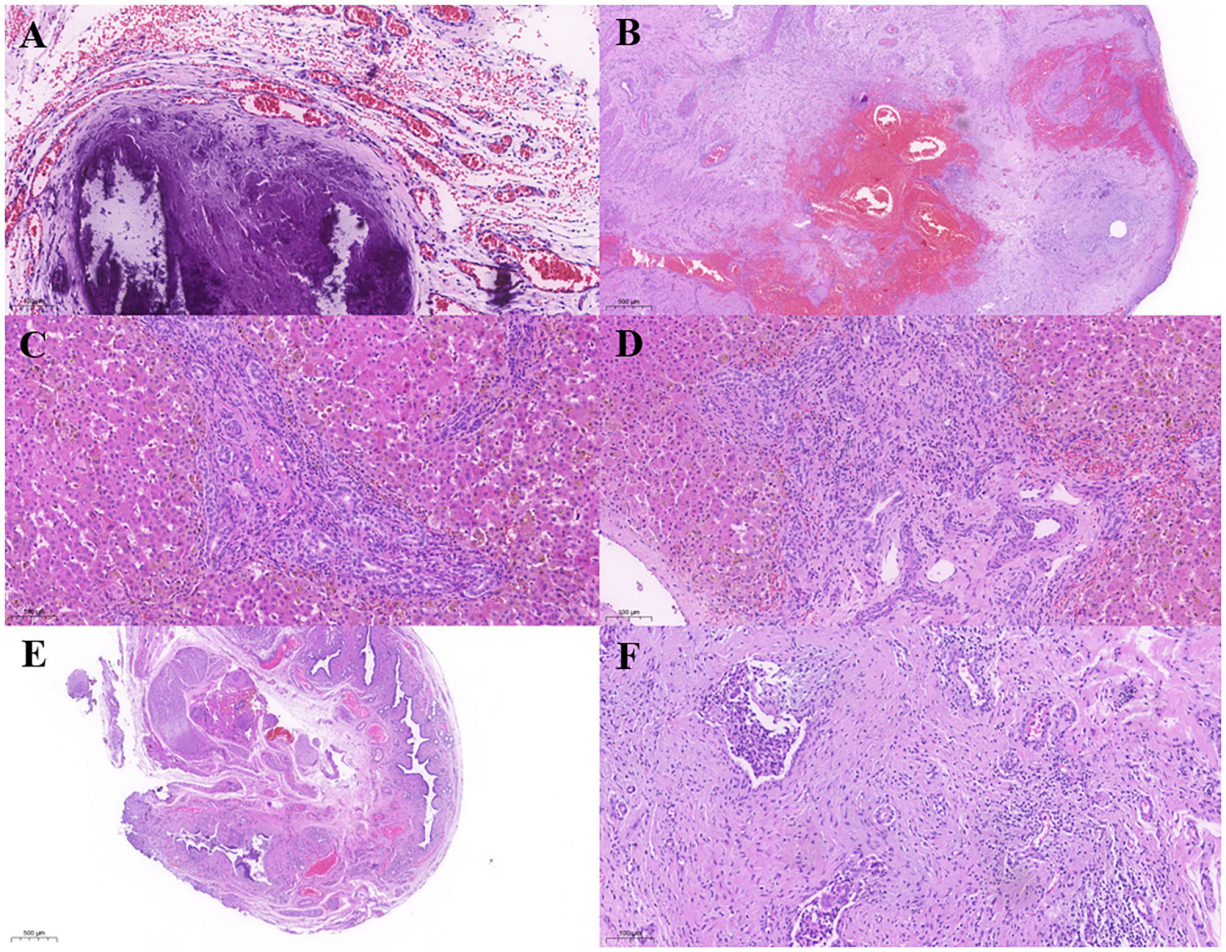
**(A)** Intestinal wall calcification. **(B)** Intestinal atresia with multinucleated giant cells. **(C,D)** Hepatic cell swelling, cholestasis, fibrosis in the portal area, inflammatory cell infiltration, small bile duct hyperplasia, vascular hyperplasia, and bile duct plate malformation. **(E)** Gallbladder atrophy, fibrous hyperplasia, surrounding bulky nerve fibers. **(F)** Fibrous tissue shows branching bile ducts and nerve fibers infiltrated with inflammatory cells.

After admission, fasting and gastrointestinal decompression were applied. A light green fluid drainage was seen out of the gastric tube. The abdomen was slightly distended with no veins showing, and the bowel sounds were hyperactive. White blood count (WBC) was 20.65^*^10^9^/L, C-reactive protein (CRP) was 0.62 mg/L; serum albumin (ALB) was 36.0 g/L, direct bilirubin (DBIL) was 5.2 μmol/L, indirect bilirubin (IBIL) was 33.1 μmol/L, alanine aminotransferase (ALT) was 17 U/L, aspartate aminotransferase (AST) was 183 U/L, gamma-glutamyltransferase (GGT) was 1,002 U/L, and total bile acids (TBA) were 8.5 μmol/L. No significant peritoneal fluid was seen on ultrasound of ascites. Liver and biliary ultrasound did not show any significant abnormalities. An upright abdominal X-ray showed signs of intestinal obstruction (Figure 1A). Emergency laparotomy revealed local intestinal adhesions and a few calcified spots. Intestinal atresia was observed at 60 cm from the ligament of Treitz. The proximal 15 cm of the hypertrophic bowel was dilated about 3–4 cm in diameter. The distal bowel was of a thin wall and 0.5 cm in diameter. The dilated 15 cm of proximal and the tiny 5 cm of distal bowel were removed, and the ileocecal part was retained. A Santullienterostomy was performed. The small bowel was measured to be 100 cm (45 cm proximal to the stoma and 55 cm distal to the stoma). Intraoperatively, no abnormal fetal stool color was found, and the gallbladder was not explored. Intraoperative diagnosis: meconium peritonitis, type IIIA intestinal atresia. An abdominal X-ray on the first postoperative day showed low bowel inflation (Figure 1B). Two days after the operation, abdominal X-ray showed a large bubble of the stomach, and hydro-aerial(HA) levels of dilated small bowel (Figure 1C). The intestine was gradually filling with air (Figure 1D). The original proximal small intestine was significantly dilated, and intestinal dynamics could not be quickly and completely restored. After surgery, the small intestine gradually inflated, and dilatation was still visible on X-rays within a short period of time. Pathology (Figures 2A,B): (ileum), the bowel is congested and edematous with localized stenosis, atresia, and calcification. A PICC tube was placed 2 days after the operation for central venous parenteral nutritional support. Jaundice was treated by phototherapy. Enteral feeding with 3 ml q3h of deeply hydrolyzed milk formula was started after 2 weeks and was gradually increased. Two weeks has passed to full feeding and gradual discontinuation of intravenous nutrition.

**Figure 3:**
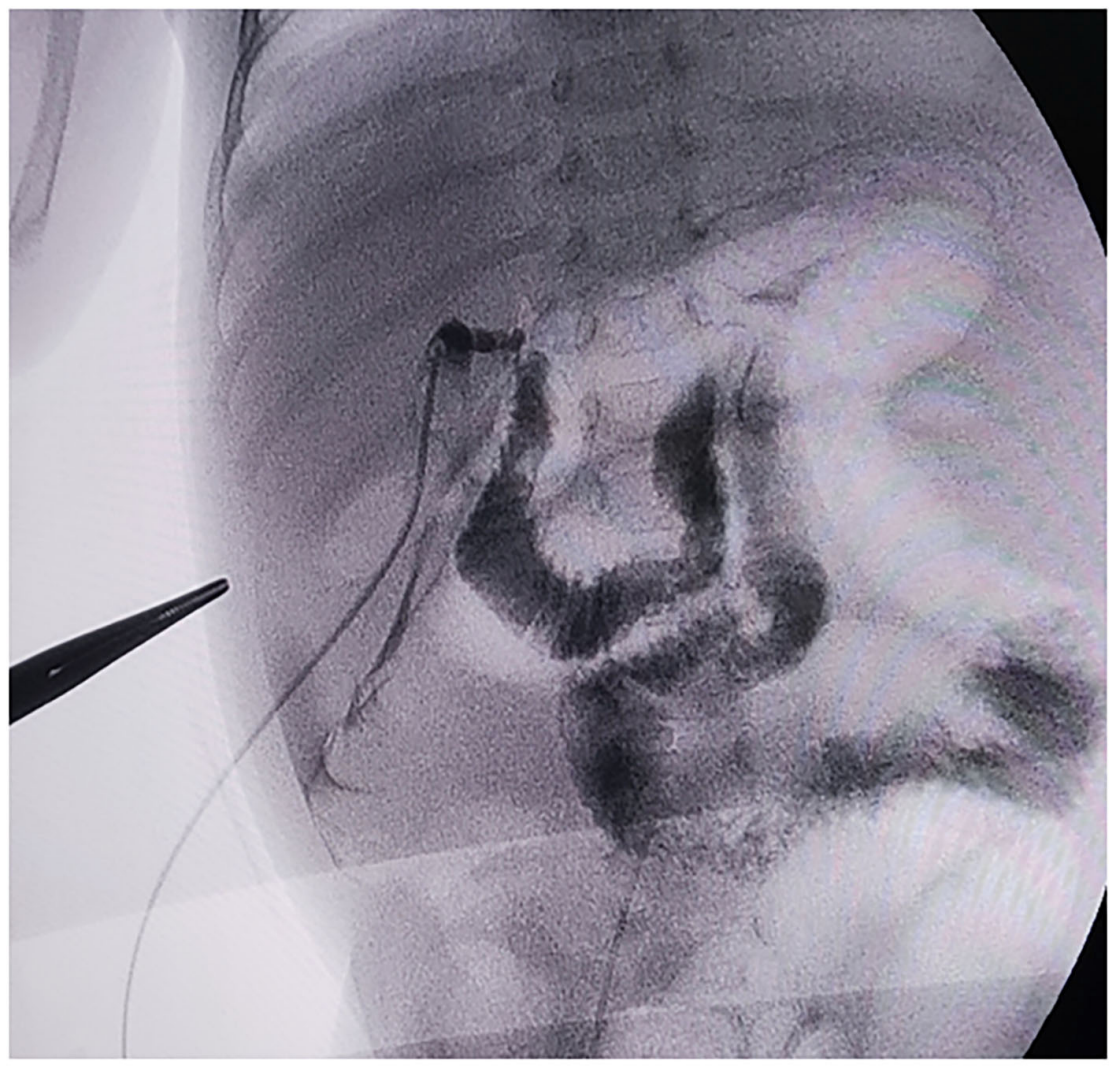
X-ray of the cholangiography during the second operation.

However, after the operation, the DBIL gradually increased, and the stool color gradually became paler from yellow-green. After the cessation of intravenous nutrition, the stool color was still white, even white clay color. Liver function index showed that the ALB was 39.1 g/L, DBIL was 149.7 μmol/L, IBIL was 104.2 μmol/L, ALT was 235 U/L, AST was 211 U/L, GGT was 610 U/L, and TBA was 135.7 μmol/L. TBA was detected by liquid chromatography-tandem mass spectrometry (LC-MS/MS) at 145.732 μmol/L, including 28.283 μmol/L for glycochenodeoxycholic acid (GCDCA) and <0.025 μmol/L for chenodeoxycholic acid (CDCA). Serum matrix metalloproteinase-7 (MMP-7) was 68.12 ng/ml. Ultrasound after 6 h of fasting showed 1.6 cm^*^ 0.4 cm of fibrous mass echogenicity in the common hepatic duct area, internal diameter of the hepatic artery was 0.27 cm with 34.9 cm/s of a peak flow velocity, RI: 0.73, internal diameter of the portal vein trunk was 0.5 cm with 25.1 cm/s of a peak flow velocity; the size of the gallbladder was 2 cm^*^ 0.5 cm. One h after feeding, no significant change in the size of the gallbladder was observed. The diagnosis of BA was confirmed during an intraoperative cholangiography procedure (Figure 3) at 46 days of age, and a KPE followed. Further surgical exploration revealed typically a solid, dense fibroinflammatory proximal remnant at the portahepatis, which was identified as type III biliary atresia. Intraoperatively, the enterostomy was closed, and the total length of the small intestine was measured at 130 cm. Because of the short total length of the small intestine, the jejunum was severed at 20 cm from the ligament of Treitz. The distal jejunum was anastomosed with the proximal jejunum at a distance of 30 cm from the severed end. The small intestine was dragged posteriorly over the colon to make a Roux-en-Y limb, and a hepatic portoenterostomy was performed. Pathology (Figures 2C–F): hepatocytes were swollen and bilious with ballooning and hepatic giant cell formation. Fibrosis in the confluent area (Ohkuma's Grade 2), inflammatory cell infiltration (Grade 2), small bile duct hyperplasia (Grade 2), bile emboli in the confluent area (Grade 1), and vascular hyperplasia (Grade 1) were seen. No bile lake formation was seen, and bile duct plate malformation was visible. The gallbladder was congested and edematous with fibrous hyperplasia, local stenosis and atresia, inflammatory cell infiltration, and scattered multinucleated foreign body giant cells. Branching bile ducts and nerve fibers with inflammatory cell infiltration were seen within the fibrous tissue. Feeding of deeply hydrolyzed formula was gradually started 3 days after surgery, and full enteral nutrition was established after 1 week. There were no intestinal motility problems and no episodic biliary tract infections during the hospitalization. The infant gained weight steadily and was discharged at the age of 2 months and 6 days.

**Figure 4:**
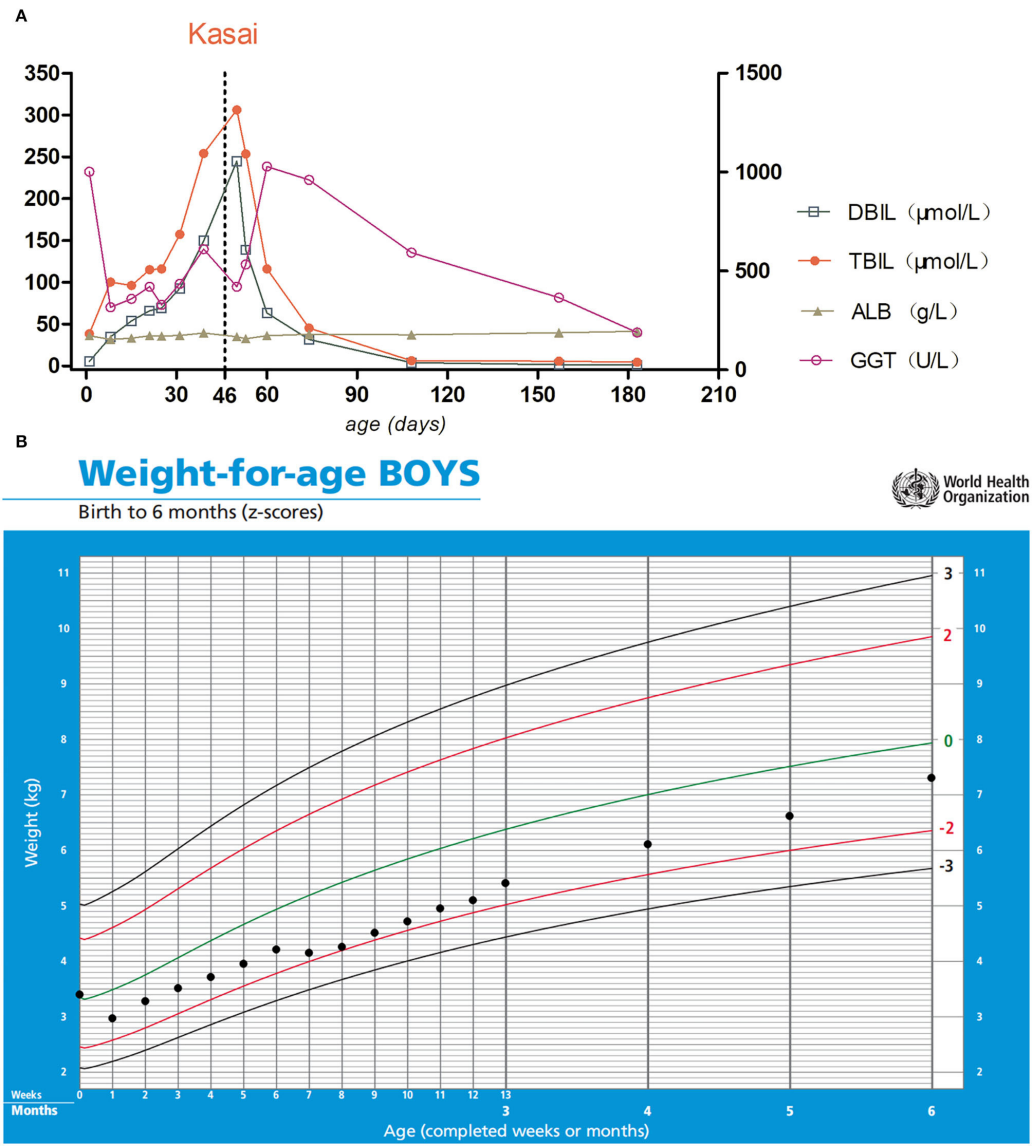
**(A)** The left Y-axis indicates DBIL, TBIL, and ALB; the right Y-axis indicates GGT. The day of Kasai's surgery is marked with the vertical dotted line. **(B)** Growth curve of the patient. Parenteral nutrition was used during hospitalization. After the enterostomy was closed, his weight gradually approached the normal range after discharge.

The bilirubin levels reached normal 2 months after KPE. At present, the follow-up to 7 months of age revealed no cholangitis, gradual improvement of liver function index (Figure 4A), and body weight showed a catch-up trend (Figure 4B). Five months after KPE, GCDCA, and CDCA were 4.487 and 0.643 μmol/L, respectively. MMP-7 was 18.37 ng/ml. The fat-soluble vitamins were all normal. Vitamin A was 583.31 ng/ml, vitamin D was 49.76 ng/ml, vitamin E was 7.16 μg/ml, and vitamin K1 was 2.09 ng/ml. Trace elements were detected as follows: magnesium, 1.49 mmol/L; copper, 15.32 μmol/L; iron, 7.55 mmol/L; zinc, 44.89 μmol/L.

## Discussion

MP, IA, and BA are all neonatal surgical conditions that require surgical treatment. Jejunoileal atresia or stenosis is a major cause of neonatal intestinal obstruction with prevalence of 1:330–1:1,500 live births. Congenital obstruction of the jejunum and ileum occurs as a result of late intrauterine vascular events, affecting the development of one or more sections of the midgut (4). MP is a type of aseptic chemical peritonitis caused by the leakage of meconium from small bowel perforations in fetuses. The incidence of MP is low, 1:30,000 live births (1). Common underlying pathologies include causes of congenital intestinal obstruction, such as small bowel atresia, midgut volvulus, Hirschsprung's disease etc. (5). Meconium ileus (MI) may be associated with IA and MP, which can be an early manifestation of cystic fibrosis (CF). CF is an autosomal recessive disease common in Caucasians, but nearly absent in Asians, which affects the respiratory tract, pancreas, gut, and hepatobiliary tract (5, 6). CF should also be considered in infants that present with white stool, especially if combined with anemia and hypoproteinemia (7). Pathology confirmed the diagnosis of BA in this case. There was no anemia, hypoproteinemia, pneumonia, diarrhea, and malnutrition during hospitalization and follow-up, and cholestasis was significantly improved. The limitation of this case report is that we did not perform a sweat chloride test and CFTR gene analysis. More than 20 years ago, Han et al. reported five cases of MP not suspected as BA during initial surgery but, later, definitively confirmed BA. Two patients died of ascending cholangitis, one patient of liver failure that was exacerbated by total parenteral nutrition (TPN)-related liver injury, one patient awaiting liver transplantation, and only one patient was in good health with no jaundice and normal growth and development (8). In this case, the patient was treated with early enteral and parenteral nutrition. BA was promptly identified by ultrasound examination, liver function index, MMP-7, and bile acid profile.

In this case, the child developed cholestasis during the enterostomy state, and, instead of immediately stopping intravenous nutrition, we switched to nutritional support with ω-3 fish oil fat emulsion. The infant was fed a deeply hydrolyzed formula containing high and medium chain triglycerides (MCT). Fat malabsorption can lead to essential fatty acid (EFA) deficiency along with impaired absorption of fat-soluble vitamins (A, D, E, and K) and trace minerals (e.g., calcium, magnesium, iron, and zinc) from BA (9). We added fat-soluble vitamins and trace minerals to intravenous nutrition to support Kasai's perioperative nutritional status.

The diagnosis of BA has been problematic, and we have supported the diagnosis based on ultrasound examination of triangular cord sign (TCS), hepatic artery diameter, long and short diameters of the gallbladder and gallbladder contraction. The TCS and gallbladder and biliary system abnormality (GBA) are two of the most widely accepted ultrasound features currently used for differential diagnosis of BA, and hepatic artery (HA) enlargement has also been used as an auxiliary sign (10, 11). Serum markers, such as DBIL, alkaline phosphatase (ALP), and GGT, are routine diagnostic indicators of BA (11). We supported BA by MMP-7 assay with values of 68.12 ng/ml preoperatively and 18.37 ng/ml 5 months post-operatively. A multicenter study conducted by Sakaguchi et al. suggested that serum MMP-7 was a useful marker for the diagnosis of BA in Japanese infants. The optimal cut-off value was 18.6 ng/ml, and the sensitivity and specificity were 100 and 90%, respectively; however, serum MMP-7 could not predict liver transplantation (LT) within a year (12). Zhao et al. (13) showed that GCDCA/CDCA can be used to assess the risk of BA in infants with cholestasis.

Most surgeons believe that a Roux loop should be 40–50 cm to produce an effective anti-reflux effect, and, if it is too short, it tends to induce postoperative cholangitis. A personalized short Roux loop of 13–20 cm (based on the distance between the hepatic hilum and umbilicus) has a comparable anti-reflux effect compared to a Roux loop of 30–40 cm in length (14). In this case, because of the short bowel, a short Roux loop was used to meet as much as possible the length of the intestinal tube to achieve total enteral nutrition. Success of KPE was defined as the clearance of jaundice and normalization of serum bilirubin 6 months after surgery (3). The infant's serum bilirubin reached the normal value 2 months after KPE surgery, and no cholangitis occurred during the follow-up period.

In this case, BA was diagnosed early by multiple examinations in the state with Santullienterostomy of the small intestine. Perioperative intravenous nutritional support was used. Shorten the Roux-en-Y limb appropriately to preserve the maximum length of the small intestine. Long-term follow-up is still needed for oral enteral nutrient absorption and cholangitis prevention. Although this is an individual case, BA should be suspected for cholestasis in infants with pale stools at the stoma.

## Data Availability Statement

The original contributions presented in the study are included in the article/supplementary material, further inquiries can be directed to the corresponding author/s.

## Ethics Statement

The studies involving human participants were reviewed and approved by Ethics Committee of Children's Hospital of Zhejiang University School of Medicine (Approval ID: 2022-IRB-083). Written informed consent to participate in this study was provided by the participants' legal guardian/next of kin. Written informed consent was obtained from the minor(s)' legal guardian/next of kin for the publication of any potentially identifiable images or data included in this article.

## Author Contributions

YH and SH conceptualized, prepared, and wrote the manuscript. BC sorted out data and made figures. SH and QQ provided the data. JT reviewed, edited, and revised the manuscript. All authors contributed to the article and approved the submitted version.

## Funding

This study was supported by a grant from the Key Program of the Independent Design Project of National Clinical Research Center for Child Health (Grant No. S20C0004).

## Conflict of Interest

The authors declare that the research was conducted in the absence of any commercial or financial relationships that could be construed as a potential conflict of interest.

## Publisher's Note

All claims expressed in this article are solely those of the authors and do not necessarily represent those of their affiliated organizations, or those of the publisher, the editors and the reviewers. Any product that may be evaluated in this article, or claim that may be made by its manufacturer, is not guaranteed or endorsed by the publisher.
